# Occurrence of *Aeromonas* Species in the Cutaneous Mucus of Barbour’s Seahorses (*Hippocampus barbouri*) as Revealed by High-Throughput Sequencing

**DOI:** 10.3390/ani13071241

**Published:** 2023-04-03

**Authors:** Rose Chinly Mae H. Ortega, Sharon Rose M. Tabugo, Joey Genevieve T. Martinez, Chinee S. Padasas, José L. Balcázar

**Affiliations:** 1 Department of Biological Sciences, College of Science and Mathematics, Mindanao State University—Iligan Institute of Technology, Iligan City 9200, Philippines; sharonrose.tabugo@g.msuiit.edu.ph (S.R.M.T.); joeygenevieve.martinez@g.msuiit.edu.ph (J.G.T.M.); chinee.padasas@g.msuiit.edu.ph (C.S.P.); 2 Molecular Systematics and Oceanography Laboratory, Premier Research Institute of Science and Mathematics (PRISM), Mindanao State University—Iligan Institute of Technology, Iligan City 9200, Philippines; 3 Mathematical Biology and Nematology Research Cluster, Complex Systems Group, Premier Research Institute of Science and Mathematics (PRISM), Mindanao State University—Iligan Institute of Technology, Iligan City 9200, Philippines; 4 Catalan Institute for Water Research (ICRA), 17003 Girona, Spain; 5 University of Girona, 17004 Girona, Spain

**Keywords:** *Aeromonas* species, Illumina sequencing, *H. barbouri*, seahorse, marine microorganisms

## Abstract

**Simple Summary:**

In this study, the bacterial community structure and composition in the skin ecosystem of Barbour’s seahorses (*Hippocampus barbouri*) were assessed by the high-throughput sequencing of 16S rRNA genes, with particular emphasis on members belonging to the *Aeromonadaceae* family due to its implications for the health of fish species. The results revealed that sequences affiliated with the *Aeromonas* genus were found in the skin of Barbour’s seahorses, with abundances being slightly similar between female and male specimens. Comparative analysis also demonstrated that the presence of *Aeromonas* species in the skin of Barbour’s seahorses was strongly influenced by the surrounding sediment. These findings could be used as a baseline for further studies about the role of *Aeromonas* species in the normal and disturbed microbiota associated with seahorses.

**Abstract:**

Although several studies have described the bacterial community composition associated with marine fish, there is limited information related to seahorses. Moreover, previous studies have demonstrated that the skin microbiota is useful for determining health status and common disorders in the host. This study, therefore, aimed to explore the skin bacterial community composition in Barbour’s seahorse (*Hippocampus barbouri*) using high-throughput sequencing of 16S ribosomal RNA genes. Water and sediment samples from the surrounding environment were also analyzed for comparative purposes. The results revealed that sequences affiliated with the *Shewanellaceae* family were dominant in the skin of female Barbour’s seahorses and sediment samples, whereas sequences affiliated with the *Bacillaceae* family were dominant in the skin of male Barbour’s seahorses. Interestingly, sequences affiliated with the *Aeromonas* genus were found in the skin of Barbour’s seahorses, whose abundance was slightly similar between the female and male specimens. Further comparative analysis showed that the presence of *Aeromonas* species in the skin of Barbour’s seahorses was strongly influenced by the surrounding sediment. Given that some *Aeromonas* species are known to be important pathogens in humans and fish, these results may be used for further research on the dependency of the skin microbial composition on the environment as well as determine whether the presence of *Aeromonas* and other detected species has implications on seahorse health.

## 1. Introduction

Although seahorses (*Hippocampus*) play important roles in the ecosystem, they are constantly at risk because of trade for medicinal and ornamental purposes [1]. Like other fish species, seahorses are intimately in contact with a complex and dynamic microbial world in which a large fraction of these microorganisms adhere to and colonize epithelial surfaces. However, in seahorses, the microorganisms are attached only to the mucous layer of a unique type of surface cell—the flame cone cells, which are found exclusively on seahorses [2]. Despite such unique features, seahorses are not exempted from diseases. In fact, some studies have reported skin diseases of diverse etiology in wild and farmed seahorses [3,4]. The fish skin microbiota possesses distinct physicochemical properties that have evolved to reside with the host and help regulate host–microbe interactions [5,6]. This regulation includes enhancement of the epithelial barrier, development of the immune system, and nutrient acquisition [7,8]. In fish species from large-scale production facilities, the mucosal microbiota is vital in protecting the host from pathogens by stimulating the immune system; however, the complex interactions of the mucosal microbiota with the pathogens and the fish skin immune system have remained unexplored [5]. In order to address these concerns, it is essential to explore the bacterial composition in the cutaneous mucus of seahorses. In a recent study carried out by our research team, we observed the presence of *Aeromonas* species in the cutaneous mucus of healthy *Hippocampus barbourin* [9]. It should be noted that *Aeromonas* species are ubiquitous bacteria primarily recovered from aquatic ecosystems [10], as they are commonly found in freshwater bodies, estuaries [11], and even in seawater [12]. Some *Aeromonas* species are typically known as opportunistic pathogens as they cause severe disease outbreaks in wild and pond-raised freshwater fish [13]. For instance, *Aeromonas salmonicida* subsp. *salmonicida* is a ubiquitous Gram-negative bacterium that causes furunculosis in wild and captive salmonids (e.g., salmon and trout). This disease results in high morbidity and mortality rates and has become a major threat to the aquaculture industry [14]. Moreover, *Aeromonas hydrophila*, commonly found in the aquatic ecosystem, is considered as an important foodborne bacterial zoonotic pathogen in aquaculture. This infectious agent is a leading cause of mortality in economically important fish species from Southeast Asia such as the striped snakehead or snakehead murrel (*Channa striata*). Despite implications for fish health [15], there is limited information on the prevalence of these bacterial species in seahorses.

Until the last decade, our knowledge of microbial communities associated with fish has largely been based on the use of traditional culture-based methods. Although useful, these data are limited and biased toward cultivable members of the community [16]. However, the study of microbial ecology has dramatically changed due to the implementation of cutting-edge sequencing technologies (such as Illumina or Oxford Nanopore), which provide new ways to study microbial communities, thereby overcoming these limitations based on culture-dependent approaches [17]. These approaches have provided a more accurate picture of microbial communities in a particular habitat following the extraction of all genetic material, followed by sequencing and bioinformatics analysis. By providing a large amount of data with high accuracy and low cost, high-throughput sequencing technology has fundamentally altered the way that research has been conducted in the past and made it possible to understand microbial diversity at a much larger scale [18]. Consequently, high-throughput sequencing technology can make an in-depth analysis of the bacterial community structure and abundance efficiently [19], not only in fish but also in other organisms. Given this, a full picture of the bacterial community composition associated with seahorses can be obtained, thereby increasing our knowledge of the role of skin microbiota in the health and disease of seahorses. This technology has therefore taken a giant leap forward in evaluating microbial communities in seahorses. In this study, we used the Illumina sequencing technology method based on 16S rRNA gene sequencing to explore the bacterial community structure and composition in the skin ecosystem of Barbour’s seahorses (*Hippocampus barbouri*), with particular emphasis on members belonging to the *Aeromonadaceae* family due to its implications for the overall health of fish species.

## 2. Materials and Methods

A gratuitous permit from the Department of Agriculture, Bureau of Fisheries and Aquatic Resources (DA-BFAR) was requested and granted under the project “Seahorses and Pipefishes with Pharmaceutical Potentials from selected areas in Mindanao, Philippines” with GP No. 0184-19. Eleven healthy Barbour’s seahorses weighing an average of 7.07 g and samples from the surrounding environment (water and sediment) were collected from coral reefs off the coast of Cantiasay Island, San Pedro, Surigao del Norte. The water had an average temperature of 23.6 °C during the sampling period. Each sample was separately and immediately transported to the Molecular Systematics and Oceanography Laboratory of the Premier Research Institute of Science and Mathematics (PRISM), Mindanao State University—Iligan Institute of Technology for further analysis. Seahorses were gently washed with sterile seawater twice to remove debris without compromising the microbial community on the skin [20]. Skin mucus samples were then collected by scraping the seahorse’s dorsal surface using a sterile swab [21], which were placed into a 2 mL microcentrifuge tube and stored at −65 °C until DNA extraction.

Genomic DNA was extracted from the skin microbial community of six female Barbour’s seahorses (HBFS) and five male Barbour’s seahorses (HBMS) as well as from water (WS) and sediment (SS) samples. Samples were pooled according to their origin and the HiPurA™ DNA Purification Kit (HIMEDIA; Mumbai, India) was used for DNA extraction according to the manufacturer’s instructions. Briefly, 1 mL of water lysis solution and samples were individually added to the Hi-Water bead tubes, which were horizontally vortexed at maximum speed for 5 min. These mixtures were centrifuged at 5000 rpm for 1 min, and the supernatants were transferred to 2 mL collection tubes and approximately 600–650 μL of each supernatant was recovered. The mixture was then centrifuged at 13,000 rpm for 1 min at room temperature and the supernatant was transferred to a new 2 mL collection tube without disturbing the pellet. A volume of 200 μL of inhibitor removal solution was added to each supernatant, and the tubes were briefly vortexed and incubated at 4 °C for 5 min. The homogenates were then centrifuged at 13,000 rpm for 1 min at room temperature, whose supernatants were transferred to 2 mL collection tubes without disturbing the pellet. A volume of 650 μL of binding solution was added to the pellet and mixed by vortexing briefly. Approximately 650 μL of this homogenate was loaded onto the HiElute Miniprep Spin Column, which was centrifuged for 1 min at 13,000 rpm. The flow-through was discarded. We repeated loading the remaining solution of 650 μL of binding solution and the pellet onto the HiElute Miniprep Spin Column and then centrifuged for 1 min at 13,000 rpm at room temperature. Then, 650 μL of diluted wash solution was added to the column and centrifuged at 13,000 rpm for 1 min. The flow-through was discarded, and then re-used the same 2.0 mL collection tube with the column. A volume of 650 μL of wash solution was added to the column and centrifuged at 13,000 rpm for 1 min. Again, the flow-through was discarded and centrifuged again at 13,000 rpm for 2 min to dry the column. The column was then placed in a new 2.0 mL collection tube and 100 μL of elution buffer was added. The solution was incubated for 5 min at room temperature, which was further centrifuged at 13,000 rpm for 1 min. The eluted DNA was then transferred to a new tube for storage at −20 °C.

Extracted DNA samples were sent to Macrogen Inc. (Seoul, Republic of Korea) for amplification, purification, quality check, and high-throughput sequencing on the Illumina MiSeq platform based on universal primers targeting the V3–V4 regions of the 16S rRNA gene. Raw data were processed using the Quantitative Insights into Microbial Ecology (QIIME 2) pipeline [22]. Operational taxonomic units (OTUs) were defined at 99% sequence similarity of the 16S rRNA genes. This value was used to define a core set of representative sequences, which were used for phylogenetic analyses. The weighted UniFrac test was applied to determine whether two or more communities had the same structure [23]. A heatmap was also generated showing the relative abundance of OTUs assigned to the *Aeromonadaceae* family across the samples, which were classified using the EzBioCloud database [24]. Phylogenetic analyses were performed by using MEGA version 6.0 [25]. Distances (distance options according to the Kimura 2-parameter model) and clustering with the neighbor-joining method were determined by using bootstrap values for 1000 replications.

## 3. Results and Discussion

**Figure 1:**
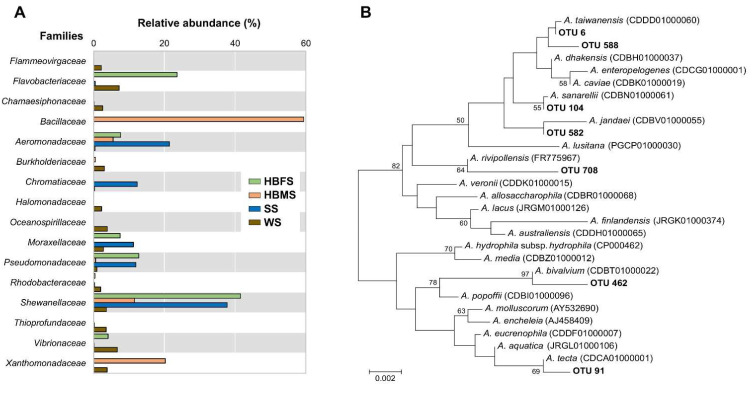
(**A**) Relative abundance of dominant bacterial families found in the skin samples from Barbour’s seahorses as well as in water and sediment samples from the surrounding environment. (**B**) Phylogenetic dendrogram of selected OTUs with the most closely related *Aeromonas* species based on 16S rRNA gene sequences (420 bp) and constructed by the neighbor-joining method. Bootstrap percentages (>50%) based on 1000 replications are shown at branch nodes. Bar, 0.002 estimated substitutions per site.

After normalizing to avoid any bias due to the difference in the total number of sequences, the bacterial community structure was analyzed using the weighted UniFrac test (sensitive to abundances of taxa), whose results demonstrated that the relative abundance of OTUs (defined at 99% similarity) was significantly different (*p* < 0.001) among groups. Overall taxonomic characterization of the bacterial community was then conducted at the family level (Figure 1A). Sequences affiliated with the *Shewanellaceae* family were dominant in the skin of female Barbour’s seahorses (HBFS) and sediment samples (SS) (41.6 and 37.8%, respectively), whereas sequences affiliated with the *Bacillaceae* family were dominant in the skin of male Barbour’s seahorses (HBMS) (59.1%). Moreover, water samples (WS) were dominated by sequences affiliated with the *Flavobacteriaceae* and *Vibrionaceae* families (7.2 and 6.7%, respectively). Although sequences affiliated with the *Aeromonadaceae* family were found in all samples, their abundance was very low in the water samples (0.4%). Specifically, sequences affiliated with the *Aeromonadaceae* family had relative abundances of 7.6 and 5.5% in the skin of female and male Barbour’s seahorses, respectively. Interestingly, a relatively high abundance of sequences affiliated with the *Aeromonadaceae* family was found in the sediment samples (21.4%). Because members belonging to this family have important implications for the overall health of fish species [26], a phylogenetic analysis was carried out to establish their taxonomic affiliation. Representative nucleotide sequences of OTUs assigned to the *Aeromonas* genus are available in Supplementary Material File S1. A phylogenetic dendrogram of selected OTUs was then constructed using the neighbor-joining method (Figure 1B), which revealed that these OTUs grouped with known *Aeromonas* species. The closest described relative of OTU 6 and OTU 588 was *A. taiwanensis* LMG 24683, whereas OTU 91 was closely related to *A. tecta* CECT 7082, OTU 104 to *A. sanarellii* LMG 24682, OTU 462 to *A. bivalvium* CECT 7113, OTU 582 to *A. jandaei* CECT 4228, and OTU 708 to *A. rivipollensis* LMG 26313. Although *Aeromonas* species share high levels of similarity based on 16S rRNA gene analysis [27], our analyses provide valuable information on the diversity of *Aeromonas* species, thereby validating the reliability of our findings. The above-mentioned *Aeromonas* species have also been observed in fishes intended for human consumption such as largemouth bass [28], sushi [29], tilapia and salmonids [30], seafood [10], and bivalve mollusks [31], whereas other *Aeromonas* species have been reported to be present in environmental samples [32,33,34,35] and in clinical samples that can be isolated from fish, and has characteristics of virulence and antimicrobial resistance that are comparable to isolates from humans [36].

Although the abundance of sequences affiliated with the *Aeromonas* genus was slightly similar between the female and male Barbour’s seahorses, there were differences in terms of diversity. In fact, the detected OTUs in the skin of female Barbour’s seahorses were affiliated with *A. bivalvium*, *A. jandaei*, *A. sanarellii*, *A. taiwanensis*, and *A. tecta*, whereas the detected OTUs in the skin of male specimens were affiliated with *A. taiwanensis* and *A. tecta*. Surprisingly, sequences affiliated with *A. taiwanensis* were highly abundant in both cases. To the best of our knowledge, this is the first time that *A. taiwanensis* has been detected in seahorses. This is of special relevance because *A. taiwanensis* was originally described on the basis of one strain recovered from the wounds of hospitalized patients in Taiwan [33]. Moreover, *A. taiwanensis* has been isolated from wastewater in Portugal [37] and from the feces of a patient with diarrhea in Israel [38].

**Figure 2:**
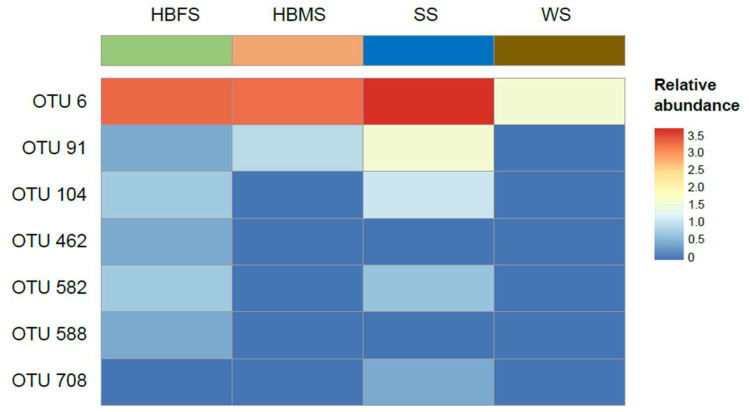
Heatmap of OTUs assigned to the *Aeromonadaceae* family, which were compared with their abundances in each sample. The color intensity (log2 scale) in each panel shows the percentage of each OTU.

It should be noted that environmental factors and microbiota composition are intimately related and have a significant impact on host health. As the skin epithelial surface is in direct contact with the surrounding environment, a comparative analysis was carried out to establish the potential influence of sediment or water on the presence and abundance of *Aeromonas* species in the skin of Barbour’s seahorses (Figure 2). The results showed that the presence of *Aeromonas* species in the skin of Barbour’s seahorses was strongly influenced by the sediments. Previous studies have suggested that the structure and composition of the skin microbiota are likely to be impacted by several variables including abiotic factors linked to the geographic locality, mineral content, temperature, and season as well as biotic factors related to the presence of other microorganisms, nutrient potential, or antimicrobial components of fish mucus [39]. Given the interface influencing this skin microbiota, water and sediments were considered [40], and in this case, the sediments had a higher influence on the skin microbiota of Barbour’s seahorses. Therefore, it appears that seahorses possess an epidermal cell type that is particularly suited for the effective adhesion of microorganisms from their environment. This is because the surface coat of the seahorse epidermis comprises several types of mucopolysaccharides: the glycocalyx, the cap of the flame cone cells, which is a mucopolysaccharide–protein complex, and the mucus secreted by unmodified cells, which resembles that of goblet cells [2]. Our findings were also supported by recent studies suggesting that the sampling site is a factor because the areas where the sediments were collected had a great impact on the skin microbial communities [41], and that the skin microbiome assemblage of marine organisms is strongly associated with the surrounding sediments [42]. In some aquacultural setups, for instance, sediments are known to be one of the primary sources of microbiota in fish, with fish microbiota also settling on the sediments [43]. This claim, therefore, provides evidence for the relationship between the microbiota composition on *H. barbouri* skin and sediments. Generally, this implies that the microbiota of marine animals including seahorses may contain significant information about how animals and the environment interact as well as about the ocean ecosystem [44].

Some studies on the microbial composition and pathogens in seahorse species have been published such as phylogenetic characterization of bacterial communities in *H. guttulatus* [45] and *H. barbourin* [9], bacterial communities in the intestinal tract of *H. kuda* (Tanu et al., 2011) [46], assessment of pathogenic bacteria from *H. erectus* [47] and *H. haema* [48]; however, no studies have been conducted on the prevalence and diversity of *Aeromonas* species in *H. barbouri*. Our findings show that the presence of *Aeromonas* species in the skin of *H. barbouri* could indicate health and disease clues. Therefore, an in-depth understanding of the dynamics and diversity of the *Aeromonas* species could provide us with reliable evidence on why they inhabit the skin mucus of *H. barbouri.* The genus *Aeromonas* is a member of the *Aeromonadaceae* family, which consists of facultatively anaerobic, Gram-negative, non-spore-forming bacilli or coccobacilli that are generally motile bacteria and commonly found in aquatic environments, some of which can cause disease in humans, fish, and other aquatic animals [49,50]. This genus comprises 36 species that are considered autochthonous of aquatic environments [51]. Numerous aquatic animals, mostly fish and corals, are involved in pathological interactions with various species of *Aeromonas* [10]. As inhabitants of marine environments, fish and other seafood are the most common sources for isolating these microorganisms [52], supporting their occurrence in seahorses. Moreover, some *Aeromonas* species are known to be opportunistic pathogens for fish. When handling fish, working in aquaculture, or keeping fish as pets, *Aeromonas* spp. can infect the skin and soft tissue, which may lead to injuries [53]. Under stressful conditions such as an increase in water temperature, poor water quality, excessive handling, etc., *Aeromonas* spp. can cause epidemic outbreaks [54]. Few known species are frequent etiological agents of fish disease such as the motile *Aeromonas* septicemia (MAS), caused by virulent *A. hydrophila, A. caviae, iA.veronii*, which is responsible for ulcerative syndrome in catfish and *A. salmonicida*, which is responsible for furunculosis in salmonids [55]. However, the severity of disease cases depends on the concentration of these microorganisms [56]. Some changes in the skin microbiota based on its phylogenetic composition may affect its functions, thereby upsetting its homeostatic interactions with the host and eventually favoring disease development [40]. Considering that *H. barbouri* specimens in this study were directly collected from the wild with no trace of skin disease, they can be considered as apparently healthy. As a result, further research is needed to understand the differences in the composition of healthy and diseased *H. barbouri*.

## 4. Conclusions 

High-throughput sequencing based on 16S rRNA amplicon sequencing technology revealed the presence of sequences affiliated with *Aeromonas* species in the skin of Barbour’s seahorses. Interestingly, *Aeromonas* species were also observed in the sediments, which seem to be the most probable source of these species. Although some *Aeromonas* species are known to be important pathogens, the presence of these species in this study may not have affected the health status of Barbour’s seahorses yet. Therefore, further studies are required to explore the implications of *Aeromonas* species and other detected species on seahorse health. 

## Data Availability

The data presented in this study are available upon request from the corresponding authors.

## Supplementary Materials

The following supporting information can be downloaded at: https://www.mdpi.com/article/10.3390/ani13071241/s1. Supplementary Material File S1: Representative nucleotide sequences of OTUs assigned to the *Aeromonas* genus.
